# Epidemiological and Economic Evaluation of a Pilot Prostate Cancer Screening Program

**DOI:** 10.1155/2020/6140623

**Published:** 2020-01-27

**Authors:** Dariga S. Smailova, Elisa Fabbro, Serik E. Ibrayev, Luca Brusati, Yuliya M. Semenova, Umutzhan S. Samarova, Farida S. Rakhimzhanova, Sabit M. Zhussupov, Zaituna A. Khismetova, Hengameh Hosseini

**Affiliations:** ^1^Semey Medical University, Semey, Kazakhstan; ^2^Department of Medicine, Udine University, Udine, Italy; ^3^Public Health Department, Astana Medical University, Nur-Sultan, Kazakhstan; ^4^Department of Economics and Statistics, Udine University, Udine, Italy; ^5^Department of Health Administration, University of Scranton, Scranton, PL, USA

## Abstract

*Background. *Prostate cancer (PCa) is the second most commonly diagnosed cancer, and the sixth most common killer among men worldwide (Aubry et al., 2013). This research was motivated by the fact that PCa screening continues to be a controversial topic in the Kazakh medical community. This study aimed at description of how newly diagnosed PCa patients are managed in Pavlodar region of the Kazakhstan Republic and at presentation of a budget impact analysis (BIA) for PCa screening program. Also, we aimed to provide a comparative analysis of pricing system on medical services applied in both private and public healthcare sectors of the Kazakhstan Republic. *Methods*. New cases of PCa have been retrospectively analyzed for the period from January 2013 to December 2017 based on the information obtained from information system “Policlinic” maintained by the Pavlodar regional branch of the Republican Center for Electronic Health and from Cancer Registry of Pavlodar Regional Oncology Center. All data were analyzed with the help of SPSS 20.0 software. *Results. *The mean age of PCa patients was 68.34 years (SD = 8.559). The government of Kazakhstan invested 20,437,000 KZT (Kazakhstani tenge) in 2017 equivalently 61,188 USD—to fund a pilot study for examination of 9638 men. From 2013 to 2017, out of 49,334 men residing in Pavlodar region of Kazakhstan 1,248 men were diagnosed with prostate diseases, including 130 PCa cases. The PCa detection rate was equal to two cases per month. Only 22.8% of all PCa cases identified in the region within specified time period were revealed as a result of the government-funded PCa screening program. The average prostate cancer detection rate among the target group of Pavlodar region within the period of 5 years was equal to 0.23%. *Conclusion. *Based on the fact that the PCa screening program failed to enable adequate detection of new PCa cases, we would not recommend to continue this type of screening unless it is undergone careful revision and replanning.

## 1. Introduction

Prostate cancer (PCa) is the second most commonly diagnosed cancer, and the sixth most common killer among men worldwide [1]. With an estimated annual incidence of 903,500 cases per year, PCa causes more than 250,000 deaths annually [2–4]. The incidence of PCa varies starkly across different regions. For example, it is well-known that the incidence rate of PCa is significantly lower in Asia than in Western countries, and much higher in the developing world than in the developed world [5]. The rates of metastatic disease and deaths from PCa in Asian countries are higher than those in Western countries due to the low exposure rate to screening [6]. PCa mortality rates have steadily decreased over the past 10 years in many countries, including England, Wales, the Czech Republic and the United States, but are increasing in Eastern European countries and in some Asian countries, such as Korea [7–9]. PCa morbidity indicators have also increased over the last several years in Kazakhstan as well [10], which may be due to introduction of the screening program that started in 2013 [11].

It is estimated that in the United States alone 1.86 billion USD are spent annually on PSA testing and more than 4 billion USD are spent annually on therapies for PCa [1]. According to 2007–2009 costs of PSA-based PCa screening, the average annual PCa screening cost per beneficiary was 36 USD. The inverse relation between beneficiary's age and screening cost was established (*p* < .001). Extrapolating the costs of the fee-for-service provided to Medicare beneficiaries (United States' healthcare for people of over 65) to population nationwide, the annual costs of PCa screening to the program were 447 million USD, including 145 million USD for men aged over 75 years [12].

Economic evaluation is particularly important for preventive medicine, which holds great potential to improve healthcare, particularly in low- and middle-income countries [13]. Studies evaluating the cost-effectiveness of PSA screening have produced a wide range of results. Despite several model-based evaluations, the robust evidence to suggest cost-effectiveness is lacking [14]. A recent study based in the United States estimated that PSA screening costs 262,758 USD per life-year gained (LYG), or over 5 million USD per death avoided [15]. A systematic review reported that cost-effectiveness is in the range of 12,000 USD /LYG to 5,000 USD /LYG, which suggests that screening is more cost-effective in men aged 50–69 years as compared to men over 70 years of age [16]. Pataky et al. argue that screening for PCa with low frequency PSA testing may be cost-effective when quality of life is not considered, but all developed 14 screening strategies had a net negative effect on the Quality Adjusted Life Years (QALYs) [17]. According to Shin et al. the national Prostate-Specific-Antigen (PSA) screening in South Korea is not cost-effective [18]. Recently, a systematic review on cost-effectiveness of PCa screening was published. Once more, this study concluded that the answer to the question of whether or not screening for prostate cancer is cost-effective remains unclear. According to existing health economics research, population-wide PSA screening is costly and ineffective but still, it may be cost-effective in certain populations [19].

Studies on the cost-effectiveness of PCa screening are primarily based on developed countries' data and relatively little is known about developing countries, especially due to the lack of large longitudinal databases [20]. The experience of Kazakhstan is interesting in this respect, since in 2013 the country launched a Prostate Cancer Screening Program which was terminated at the end of 2017. This decision could be partly attributed to the lack of reliable PCa screening system in Kazakhstan. In fact, the results of a recently published study indicate that PSA test is not a reliable method for identification of PCa among Kazakhstani patients as there was a high ratio of false-positive results for this test, which resulted in unnecessary biopsies. This 2017 study also found that the test's sensitivity is as high as 96.61%, but the specificity is as low as 10.43% [21]. This study aimed at description of how newly diagnosed PCa patients are managed in Pavlodar region of the Kazakhstan Republic and at presentation of a budget impact analysis (BIA) for PCa screening program. Also, we aimed to provide a comparative analysis of pricing system on medical services applied in both private and public healthcare sectors of the Kazakhstan Republic.

## 2. Materials and Methods

In Kazakhstan, the healthcare system essentially focuses on the provision of primary care. This goes back to the Soviet days, to the Alma-Ata 1978 International Conference on Primary Healthcare, or Health for all, which emphasized the significance of primary care. This conference is famous for the adoption of the so-called Alma-Ata Declaration. Kazakhstan is currently implementing the state program for the development of healthcare system entitled “Densaulyk”, which covers the period of 2016–2019. Based on this program, the government of Kazakhstan pays particular attention to the provision of national screening programs. Concerning PCa screening, it was carried out in the period from 2013 through 2017, and covered 11 out of the 16 country regions. The country introduced government-funded serum PSA testing among men aged 50, 54, 58, 62 and 66 years. In 2013, the pilot PCa screening were initiated in East Kazakhstan, West Kazakhstan, Kyzylorda and Pavlodar regions, as well as in two cities of national significance (Almaty and Astana). In 2014, this effort was extended to Aktobe, Atyrau, Karaganda, Kostanay and North Kazakhstan regions [10]. However, since 2018, the government-funded PCa screening has been stopped due to the expansion of target age groups for breast, cervical and colorectal cancers.

All PCa screening procedures were regulated by the Order of the Ministry of Health “On approval of the Rules for conducting preventive medical examinations of target population groups” of November 2009 (N 685). According to this Order, the target population groups were men aged 50, 54, 58, 62 and 66 years not followed for prostate cancer. The primary healthcare establishments invited all men of appropriate ages by means of direct contact, phone calls or via family members/neighbors to attend the clinic for a PSA blood test. For the PCa screening within 2013–2017, 49,334 agreed to participate. According to the local health plan, in 2017 out of 20,628 men aged 50, 54, 58, 62 and 66 years, only 9,638 men took part in the Prostate Screening Program, which resulted in 46.7% response rate. Besides testing serum PSA levels by means of immunohistochemistry, the PCa screening also involved evaluation of the so-called “Prostate Health Index” (PHI) that was based on measurements of total PSA, free PSA and proPSA. The PSA cut-off for a prostate biopsy was total PSA ≥7.8 ng/ml or PHI ≥25. The actual number of men who had undergone prostate biopsy was not reported.

Pavlodar region was involved in this pilot screening program for PCa within the entire period of its implementation, which equaled five years. The region locates in North-Eastern part of Kazakhstan and has a population of 769,346 people. Of these, 49,334 men were exposed to PCa screening from January 2013 to December 2017 that was organized in primary healthcare centers throughout the region. General practitioners and nurses drove data about participants' cases into the screening registry. Afterwards, all cancer cases identified were registered in the cancer registry by the staff from the local oncology center. We obtained this dataset and subjected it to retrospective analysis. The dataset included all prostate cancer cases that were diagnosed between January 1, 2013 and December 31, 2017 (code C61, based on the International Classification of Diseases, 10^th^ edition). For economic analysis, we obtained the official financial documents from the Healthcare Department of Pavlodar region. Information about prices on medical services of private sector was analyzed based on the data presented on official websites of health care establishments.

All patients with verified PCa undergo treatment at governments expense. The available treatment options are detailed in the national guidelines “Cancer of the Prostate” that follow recommendations given in Clinical Practice Guidelines of the European Society for Medical Oncology (https://www.esmo.org/Guidelines/Genitourinary-Cancers/Cancer-of-the-Prostate).

### 2.1. Statistical Analyses

IBM SPSS Statistics version 20.0 was used for all data analyses. We computed the following variables: age, stage of the disease, year of diagnosis, place of residence and PCa cases detected out of screening program. Student's t-test, Pearson's chi-square test and one-way analysis of variance were applied to test for the difference. Student's *t*-test was used to compare the mean values of continuous variables, like age and residence, while comparison between the mean values of variables like disease stage/year of diagnosis were conducted by the one-way analysis of variance. Chi-squared test was used to compare the categorical or nominal variables. Normally distributed data were expressed as the mean and standard deviation. Differences were considered statistically significant when *P* ≤ 0.05.

The survey was approved by the Ethical Committee of Semey Medical University, Kazakhstan.

## 3. Results

Overall, there were 49,334 men who were screened for PCa within 2013–2017 by means of serum PSA measurement. Of these, 47,234 men had PSA level not exceeding 3.1 ng/ml, in 1,679 men the PSA levels ranged from 3.1 to 7.8 ng/ml, and in 421 men the serum PSA was 7.8 ng/ml and higher. There were 2.5% of men who were diagnosed with benign prostate hyperplasia or prostatis following the subsequent evaluation (Table 1).

Out of 49,334 men screened in Pavlodar region, only 130 (0.26%) were diagnosed with PCa within the framework of screening program carried from 2013 to 2017. However, the overall number of newly diagnosed PCa in Pavlodar region within the same time period is equal to 568 cases, which means that only 22.8% of these were identified due to the government-funded PCa screening program. In this study the annual PCa detection rate increased modestly from 0.1% in 2013 to 0.4% in 2017, and the average 5-year PCa detection rate in the target population group was 0.26% (Table 2).

The further characteristics of identified prostate cancer cases are shown in Table 3. The major finding is the gradual annual increase of PCa cases. Most of PCa cases were diagnosed at the second stage (67.8%), which was significant (*p* < 0.001). The mean age of PCa patients was 68 years (M = 68.34 (95% CI: 67.64–69.05) SD = 8.559), while the minimum age was 41 years and the maximum age was 92 years, Me = 67. Most PCa cases were registered among urban residents of Pavlodar region (76.2%). The mean age of PCa patients residing in urban area was 68.88 years (SD = 8.260), and the mean age of rural PCa patients was equal to 66.62 years (SD = 9.350).

As PCa screening was provided to men aged 50, 54, 58, 62 and 66 years, it was not surprising to see that most PCa cases were identified in men of corresponding ages. Out of 568 patients identified within 2013–2017, most prostate cancer cases (271) were in the age range from 50 to 66 years and Table 4 presents more details on this category of individuals.

Economic evaluation of PCa screening services is presented in Tables 5 and 6.  Table 5 shows the average prices of prostate cancer diagnostics in private and public healthcare sectors of the Kazakhstan Republic. In general, the prices are much higher in private healthcare sector as compared to the public healthcare sector.

As it is seen in Table 5, prostate biopsy with histological evaluation was the only type of service that was cheaper in the private healthcare sector, as compared to the public one. Pro-PSA levels testing was only provided by the public healthcare establishments.

Prostate Cancer screening costs in Pavlodar region in 2017 is shown in Table 6. There are 4 stages of screening program with total financing of screening program and costs for medical services according to republican tariff: venous blood sampling total PSA, Free PSA, Pro-PSA, biopsy, Transrectal Ultrasonography of the Prostate, urologist's consultation, histology. At the first stage of screening program 12,755,000 tenge (38,188 USD) was invested for venous blood sampling and total PSA determination. At the second stage of screening program 4,006,000 tenge (11,994 USD) was invested for determination of total PSA, free and Pro-PSA levels. Prostate biopsy per 1 patient costs 5,116 Kazakhstani tenge equivalent of 15 USD. In 2017 biopsy was taken from 145 patients which cost 742,000 tenge equivalent to 2,221 USD.

## 4. Discussion

To the best of our knowledge, this study is the first Kazakhstani study to combine economic and epidemiological assessment of a pilot screening program for PCa in Pavlodar region of the Kazakhstan Republic. The major finding is rather disadvantageous as only 22.8% of all PCa cases identified in the region within specified time period were revealed as a result of the government-funded PCa screening program. To compare this finding with other world countries, we could make a sample of Finland, where up to 46% of PCa cases were identified outside the screening program. As for Sweden and Netherlands, the proportion of PCa cases identified outside the screening program was just 20%. Probably, this remarkably low proportion of patients identified outside the screening program could serve as a quality indicator [22]. In general, this has to be noted when recommendation to screen or not to screen is concerned. Any type of screening program should be based on a positive balance between the benefits (early detection of high-risk PCa, and eventually a mortality reduction) and harms (unnecessary testing/biopsies and overdiagnosis/overtreatment of indolent disease) of screening. Also, finding a reasonable balance between benefits and harms is essential for improved patients' satisfaction with the quality of healthcare services provided [23, 24]. It might be concluded that the PCa screening program in Pavlodar region of Kazakhstan failed to achieve the international standards because as many as 77.2% of PCa cases were identified outside the screening program.

Several assumptions could be made to explain this phenomenon. First, the pilot screening program was initially targeted on men aged 50, 54, 58, 62 and 66 years, while the older is the man, the higher is his risk to develop prostate cancer (American Cancer Society: Cancer Facts and Figures 2017. Atlanta, Ga: American Cancer Society, 2017). So, this low quality indicator could be partially explained by simple exclusion of other age groups. Second, the response rate for prostate cancer screening was 46.7%. Third, the PSA cut-off value for prostate biopsy was 7.8 ng/ml and as the actual number of men who received a biopsy was not reported, the positive predictive value (PPV) for prostate cancer remains unclear. Still, the PSA cut-off for biopsy used in Kazakhstan could be considered as very high according to international standards, which are commonly based on a PSA cut-off of >3.1 ng/ml [25, 26]. Presumably, this fact is the most important cause of the low prostate cancer prevalence (0.26%) identified by our study. Since a total of 4% (or 2,100 out of 49,334) of men had a PSA >3.1 ng/ml, this yields a PPV of 25–33% as reported by most screening studies. Because only 0.9% (or 421 out of 49,334) of men were eligible for prostate biopsy based on the criteria applied in Kazakhstan (PSA >7.8 ng/ml), the PCa screening program failed to achieve the international standards. Finally, it might also be concluded that entire PCa screening program had room for improvement in terms of establishing effective recruitment, selection and referral mechanisms.

However, in our study the PCa detection rate increased gradually from 0.1% in 2013 to 0.4% in 2017, while the average detection rate of PCa was 0.23% within the period of five years. Nevertheless, a recommendation against (or for) prostate cancer screening solely based on the detection rate (and the related costs), does not seem to be fair. Japan is another Asian country and it has the annual PCa detection rates within the range of 0.54–1.13%, which is very similar to our findings [27]. To date, PCa screening remains one of the most controversial topics for public health policy [28]. To make it more effective, a number of approaches have been proposed. It was recommended to avoid the usage of PSA testing in previously screened men aged less than 55 years [29], also in men older than 70 years of age, and in all men with a short life expectancy or with serious comorbid conditions [30].

Basically, the value of PSA testing for early detection of PCa remains a subject of robust debates in medical and popular literature [31]. For instance, the age-adjusted incidence rates of de novo metastatic PCa were reported to decrease by 65% between 1988 and 2009. This well-demonstrated stage migration of PCa is said to be the direct result of widespread PSA screening over this time span [32]. However, on the other hand, according to some studies PSA test alone has low predictive value, since it may give rise to many false positive results that leads to unnecessary biopsies and over diagnosis [33]. Researchers from School of Medicine at the University of Alabama at Birmingham developed a PSA-age volume (AV) score by multiplying the patient age by the prostate volume and dividing it by PSA level. According to their data, PSA-AV scores showed to be useful for predicting positive biopsy findings [34]. According to the data of Chinese researchers, the PSA-AV scores performed well with prostate specific antigen density (PSAD) and were better than PSA alone in predicting PCa, suggesting that PSA-AV scores could be useful for predicting PCa in Chinese population, especially among younger patients and patients with small prostates [4]. Several Turkish researchers confirmed that PSA-AV cut-off of 700 could be used for predicting positive prostate biopsy findings in patients under the age of 60, and in those with low prostate volumes [35].

We think that it was the unique opportunity for Kazakhstani population to have PCa screening absolutely for free. The budget impact analysis showed that the Ministry of Health of the Kazakhstan Republic invested 27 million Tenge (approximately 178,656 USD) to screen 9953 men residing in Pavlodar region in 2013; 20 million Tenge to screen 9638 men residing in Pavlodar region in 2017.

In other words, with two cases of PCa detected every month has helped to test 49,334 men and diagnose 1,248 prostate disease, including 130 PCa cases. Taking into account the fact that most cases (67.8%) were identified at the Stage II, and as little as 4.9% of cases were identified at Stage I, this investment appears to be rather doubtful.

The comparison of healthcare sector's costs is best between the countries sharing similar political and economic background. For Kazakhstan, such countries are the countries of the former Socialist block. Unfortunately, we failed to identify such studies even with the careful search of information in grey literature databases. For this reason, we can only rely on available publications, which mostly come from the developed world nations.

To compare the costs for detection of PCa between the Kazakhstan Republic and other world countries, the conclusion could be drawn that these are dramatically higher in the countries with established market economies. For example, in Ontario (Canada) the cost of a PSA test is 30 Canadian dollars (CAD), which is 22.7 USD or 8,400 KZT, and the cost of urologist's visit is 80 CAD (60.7 USD or 22,402 KZT) [36], whereas in Kazakhstani prices one urologist's visit is 1.87 USD (692.44 KZT) in public sector and 8.14 USD (3000) in private sector. Based on these figures, a population-based PSA screening program for men aged 50–74 years in Ontario would cost approximately 149.4 million USD per year assuming that 52% of Ontario males in that age group are participating [36]. As for realities of Kazakhstan, the PCa pilot screening program for men aged 50, 54, 58, 62, and 66 years residing in Pavlodar city of Pavlodar region have resulted in the costs of 77,038 USD as of 2013. So, it is not surprising to see that screening costs in Kazakhstan were much lower as compared to the developed world countries. In general, screening costs are obviously low in Kazakhstan as it is a developing country, but still these numbers appear to be pretty high taking into account overall health expenditure (HE) per capita in the country. Such, according to the World Bank, HE in Kazakhstan accounted for about 4.4% of Gross Domestic Product (GDP) and in 2014 was equal to 539 USD per capita, while in Canada HE accounted for 10.4% of total GDP and was equal to 5,292 USD per capita in the same year.

Certainly, this study is not fully representative of the entire country as local variations in PCa rates may exist. In fact, this study could be declared as a pilot and might serve as the first step for further implementation of large-scale epidemiological studies on the whole country, bringing data from other regions that were covered by PCa screening program together and comparing them with those regions that were not participating in the screening. Understanding the rates of PCa is an important task for public health professionals, general practitioners, and oncologists as they are essential for developing programs targeted on reduction of PCa mortality and improvement of survival rates.

## 5. Conclusion

The present study is a 5-year retrospective study, which aimed to provide a descriptive analysis on newly diagnosed PCa cases detected in Pavlodar region of the Kazakhstan Republic following implementation of the PCa screening program. As a part of our analyses we presented the BIA for PCa screening program and a comparative analysis of pricing system on medical services provided by the public and private healthcare sectors of the Kazakhstan Republic. In general, the detection rate of PCa was rather low and did not achieve that reported by other studies. Still, there was a gradual increase in PCa detection rates within the study period. Consequently, the Ministry of Health, Kazakhstan increased financing for PCa screening in Pavlodar region following the inflation rates. Although the costs of PCa screening in Pavlodar region were lower than those reported by the developed world nations, they could be considered as rather substantial taking into account overall HE in Kazakhstan. Based on this fact and also on the failure of this program to enable adequate detection of new PCa cases, we would not recommend to continue this type of screening program unless it is undergone to careful revision and replanning. Any screening program should be balanced appropriately to avoid unnecessary testing and overdiagnosis, while to promote early detection of high-risk cancers, which will eventually result in mortality reduction.

## Figures and Tables

**Table 1:** Serum PSA levels, free PSA, ProPSA, and PHI in men of Pavlodar region screened for PCa within 2013–2017.

Year	Number of patients examined for PSA	Results of PSA test			Free PSA & ProPSA	PHI 25 and above	Number of identified diseases (N40, N41)	Number of identified PCa		
		<3.1 ng/ml	3.1–7.8 ng/ml	>7.8 ng/ml				2–4 points according to Gleason	5–7 points according to Gleason	8–10 points according to Gleason
2013	9888	9504	331	53	129	91	196	8	5	0
2014	11666	11250	320	96	265	200	279	16	6	8
2015	9322	8877	367	78	268	151	355	4	1	2
2016	8821	8426	322	73	188	153	204	4	5	11
2017	9637	9177	339	121	185	142	214	4	6	9

Total	49334	47234	1679	421	1035	737	1248	36	23	30

**Table 2:** Main indicators of the pilot PCa screening program in Pavlodar region, 2013–2017.

No.	Indicator	Year					Total for 5 years
		2013	2014	2015	2016	2017	2013–2017
1	Number of men planned for the screening	9953	11664	9327	8808	9638	49390
2	Actual number of men screened	9888	11666	9322	8821	9637	49334
3	Plan implementation, %	99.35	100.02	99.95	100.15	99.99	99.84
4	Number of patients detected with prostate disease^∗^	196	279	355	204	214	1248
5	Actual prostate disease detection rate, %	1.98	2.39	3.82	2.31	2.22	2.53
6	Planned prostate disease detection rate, %	1.97	2.39	3.81	2.32	2.22	2.53
7	*The number of prostate cancer cases identified due to a pilot screening program*	13	30	17	31	39	130
8	I Stage	6	3	1	0	2	12
9	II Stage	5	24	13	28	35	105
10	III Stage	2	3	2	3	2	12
11	IV Stage	0	0	1	0	0	1
12	The proportion of prostate cancer cases identified within the framework of pilot screening program	0.1	0.3	0.2	0.35	0.40	0.26
13	The proportion of early PCa cases identified within the framework of pilot screening program	84.62	90.00	82.35	90.32	94.87	90
14	The proportion of late PCa cases identified within the framework of pilot screening program	15.38	10.00	17.65	9.68	5.13	10
15	*The number of newly diagnosed prostate cancer cases (including those identified due to the pilot screening program)*	95	99	101	148	125	568
16	I stage	5	8	7	3	5	28
17	II stage	55	59	65	107	99	385
18	III stage	21	20	20	31	6	98
19	IV stage	14	12	9	7	15	57
20	The proportion of prostate cancer cases identified at an early stage	63.16	67.68	71.29	74.32	83.20	72.71
21	The proportion of prostate cancer cases identified at a late stage	36.84	32.32	28.71	25.68	16.80	27.29
22	The proportion of newly diagnosed prostate cancer cases identified by the pilot screening program out of the total number of newly diagnosed cases of prostate cancer	13.68	30.30	16.83	20.95	31.20	22.89
23	Total number of newly diagnosed cancers of all types	2,152	2,272	2,248	2,373	2,448	11,493
24	The proportion of prostate cancer cases out of all cancer cases	4.41	4.36	4.49	6.24	5.11	4.94

^∗^Prostate disease is any prostate disorder that results in elevated PSA levels.

**Table 3:** Frequency of prostate cancer cases by year of identification, cancer stage and area of residence at the time of diagnosis: Pavlodar region, 2013–2017.

Variable	Frequency (%), *n* = 568	Mean age (SD)	*P*-value
*Year*			
2013	95 (16.7)	67.39 (7.422)	*p* = 0.025
2014	99 (17.4)	67.62 (7.603)	
2015	101 (17.8)	70.53 (8.827)	
2016	148 (26.1)	68.86 (9.278)	
2017	125 (22)	67.26 (8.725)	

*Stage*			
1	28 (4.9)	66.25 (7.773)	*p* = 0.159
2	385 (67.8)	68.12 (8.170)	
3	98 (17.3)	68.63 (9.634)	
4	57 (10)	70.35 (9.349)	

*Residence*			
Urban	433 (76.2)	68.88 (8.260)	*p* = 0.005
Rural	135 (21.8)	66.62 (9.350)	

Total	568	68.34 (8.559)	

**Table 4:** Frequency of PCa cases identified in men aged 50–66 years: Pavlodar region, 2013–2017.

Age	**50**	51	52	53	**54**	55	56	57	**58**	59	60	61	**62**	63	64	65	**66**	**50–66**
*n*	**5**	2	0	0	**10**	6	6	6	**36**	9	8	5	**54**	12	14	12	**86**	**271**
%	**0.9**	0.4	0	0	**1.8**	1.1	1.1	1.1	**6.3**	1.6	1.4	0.9	**9.5**	2.1	2.5	2.1	**15.1**	**47.9**
*χ* ^2^ test of difference	41.986				30.292				0.184				9.509				87.034	2.3803
*p*-value	<0.001				<0.001				0.668				0.003				<0.001	0.1229

**Table 5:** The average prices of public and private healthcare sectors to diagnose a case of PCa in the Republic of Kazakhstan.^∗^

Type of service provided	Price per one patient, KZT (USD)	
	Public	Private
Venous blood sampling	92.9 (0.25)	400 (1.08)
Total PSA levels testing	1121.43 (3.04)	2500 (6.78)
Free PSA levels testing	2022.94 (5.48)	2500 (6.78)
Pro-PSA levels testing	24,341.34 (66.05)	-
Urologist consultation	692.44 (1.87)	3000 (8.14)
Transrectal ultrasonography of the prostate	569.7 (1.54)	3500 (9.49)
Prostate biopsy & histological evaluation	6986.86 (18.95)	6500 (17.63)

^∗^Source: Department of Healthcare of Pavlodar region and official websites of private clinics, 2017.

**Table 6:** Prostate cancer screening costs in the Pavlodar region in 2017.

Stages/year	1 Stage of screening			2 Stage of screening			3 Stage of screening			4 Stage of screening			Total KZT (thousand)
	Number of target patients aged 50, 54, 58, 62, 66 for total PSA	Sum per 1 patient, KZT	Total sum, KZT (thousand)	Number of patients for free & ProPSA (4%)	Sum per 1 patient, KZT	Total sum, KZT (thousand)	Number of patients for biopsy (3%)	Sum per 1 patient, KZT	Total sum, KZT (thousand)	Number of patients for histology (3%)	Sum per 1 patient, KZT	Total sum, KZT (thousand)	
*Total*	**9638^∗^**	**1323,42^∗∗^**	**12755^∗∗^**	**145**	**27625,05^∗∗^**	**4006^∗∗^**	**145**	**6561,0^∗∗^**	**951^∗∗^**	**145**	**2348,7^∗∗^**	**2724^∗∗^**	**20437**
*p*-value	<0.001	<0.001	<0.001	<0.001	<0.001	<0.001	<0.001	<0.001	<0.001	<0.001	<0.001	<0.001	<0.001
*1 Stage*	**9638**	**1323,42**	**12755**										**12755,1**
Venous blood sampling	9638	134,02	1292										1291,7
Total PSA levels testing	9638	1189,4	11463										11463,4
*2 Stage*				**145**	**27625,05**	**4006**							**4005,6**
Total PSA levels testing				145	1189,4	172							172,5
Free PSA levels testing				145	2091,05	303							303,2
Pro-PSA levels testing				145	24344,6	3530							3530,0
*3 Stage*							**145**	**6561,00**	**951**				**951,3**
Urologist consultation							145	770,85	112				111,8
Transrectal Ultrasonography of the Prostate							145	673,71	98				97,7
Prostate Biopsy							145	5116,44	742				741,9
*4 Stage*										**145**	**2348,67**	**2724**	**2724,5**
Histological evaluation										145	2348,67	2724	2724,5

^∗^ *χ* ^2^ test of difference. ^∗∗^ANOVA. The chi square test is only valid for comparing the number of patients at the first stage of screening with subsequent screening stages. We did not compare the subsequent screening stages between each other as these are the same observations.

## Data Availability

All the data to make the conclusions is included in the article.

## Abbreviations

LYG: Life-year gained
HE: Health expenditure
PCa: Prostate cancer
PPV: Positive predictive value
PSA: Prostate-specific antigen
PSA-AV: Prostate-specific antigen-age volume
QALYs: Quality adjusted life years.
